# A *Phytophthora sojae* effector PsCRN63 forms homo-/hetero-dimers to suppress plant immunity via an inverted association manner

**DOI:** 10.1038/srep26951

**Published:** 2016-05-31

**Authors:** Qi Li, Meixiang Zhang, Danyu Shen, Tingli Liu, Yanyu Chen, Jian-Min Zhou, Daolong Dou

**Affiliations:** 1 Department of Plant Pathology, Nanjing Agricultural University, Nanjing 210095, China; 2 Center for Genome Biology and State Key Laboratory of Plant Genomics, Institute of Genetics and Developmental Biology, Chinese Academy of Sciences, Beijing 100101, China

## Abstract

Oomycete pathogens produce a large number of effectors to promote infection. Their mode of action are largely unknown. Here we show that a *Phytophthora sojae* effector, PsCRN63, suppresses flg22-induced expression of *FRK1* gene, a molecular marker in pathogen-associated molecular patterns (PAMP)-triggered immunity (PTI). However, PsCRN63 does not suppress upstream signaling events including flg22-induced MAPK activation and BIK1 phosphorylation, indicating that it acts downstream of MAPK cascades. The *PsCRN63*-transgenic *Arabidopsis* plants showed increased susceptibility to bacterial pathogen *Pseudomonas syringae* pathovar *tomato* (*Pst*) DC3000 and oomycete pathogen *Phytophthora capsici*. The callose deposition were suppressed in *PsCRN63*-transgenic plants compared with the wild-type control plants. Genes involved in PTI were also down-regulated in *PsCRN63*-transgenic plants. Interestingly, we found that PsCRN63 forms an dimer that is mediated by inter-molecular interactions between N-terminal and C-terminal domains in an inverted association manner. Furthermore, the N-terminal and C-terminal domains required for the dimerization are widely conserved among CRN effectors, suggesting that homo-/hetero-dimerization of *Phytophthora* CRN effectors is required to exert biological functions. Indeed, the dimerization was required for PTI suppression and cell death-induction activities of PsCRN63.

Plants make use of two tiered innate immunity to fend off microbial infection. The first layer is triggered upon the perception of pathogen-associated molecular patterns (PAMPs) by pattern-recognition receptors, and thereafter termed PAMP-triggered immunity (PTI). The second layer is effector-triggered immunity (ETI) that is initiated upon the perception by intracellular immune receptors of pathogen effectors delivered into the host cell^1^. Successful pathogens are able to overcome PTI and even ETI by producing secreted effectors^2^,^3^. This arms race between the plant surveillance system and pathogen effectors was proposed as a “zig-zag model”^1^. PAMPs are often conserved among different classes of microbes and have essential functions in microbial fitness or pathogenicity. At least six different groups of PAMPs have been identified and characterized in oomycete pathogens that belong to the kingdom of Stramenopila and contain many notorious pathogens, such as *Phytophthora sojae* and *P. infestans*^4^. These PAMPs include the heptaglucoside fragments originating from branched β-glucans in cell wall^5^, a 13-aa peptide derived from the calcium-dependent cell wall transglutaminase (TGase)^6^, elicitins with sterol-binding activity (e.g. *P. infestans* INF1)^7^, cellulose binding elicitor lectin and the conserved peptide fragments of Nep1-like proteins^8^,^9^, and a glycoside hydrolase family 12 protein (XEG1)^10^. All these molecules are widely distributed and strongly conserved in oomycete pathogens and may activate plant immune responses. Thus, the pathogens were assumed to develop large amounts of intracellular effectors to suppress PTI during co-evolution^11^,^12^.

Among the oomycete intracellular effectors, the RXLR (R represents arginine, L represents leucine and X is any amino acid) and CRN (crinkler or crinkling- and necrosis-inducing protein) effectors are two utmost important groups^3^. These effectors are modular proteins; their N-terminal are conserved and responsible for delivering proteins into hosts plant cells^13^,^14^,^15^, while the C-terminal parts are relatively diverse and function inside host cells to manipulate plant immunity responses^16^,^17^. It is usually difficult to predict their functions and mechanisms because of a lack of sequence similarity to known proteins. Functional characterizations of these intracellular effectors indicated that about half of them may suppress INF1-triggerrd cell death in plants^18^,^19^. For instance, *P. infestans* Avr3a may target and stabilize plant U-box E3 ligase CMPG1 to prevent INF1-mediated cell death specifically and CMPG1 is an essential component in INF1-induced immunity^20^.

Recognition of oomycete PAMPs and signaling pathway in plants are still being uncovered. Analysis of plant genes regulated by HaNLP3, a *Hyaloperonospora arabidopsidis* Nep1-like protein derived PAMP, showed that there was a strong overlap with genes up-regulated in response to a well-studied bacterial PAMP, flg22^9^,^21^. Flg22 is a conserved 22- amino acid widely found in flagellin, the filament subunit of the bacterial flagellum^22^. It is directly recognized by plant FLAGELLIN SENSITIVE2 (FLS2) and then instantly mediates association between FLS2 and BRI1-associated receptor kinase 1 (BAK1) to form a signaling-activate complex^23^,^24^. And finally, the plant immunity is triggered and numerous defense-related genes are induced by activating a downstream mitogen activated protein kinase (MAPK) pathway. Among them, *FRK1* (*FLG22-INDUCED RECEPTOR-LIKE KINASE 1*) has been widely used as a reporter gene of PAMP-induced responses^12^,^25^. Many bacterial effectors appear to suppress flg22-triggered immunity and block the expression of defense-associated genes with distinct mechanisms. The *Pseudomonas syringae* effectors AvrPto/AvrPtoB target the pattern recognition receptor complex^26^,^27^,^28^, and *P. syringae* effectors HopAI1 and HopF2 target plant MAP kinase cascade^29^,^30^, while *Xanthomonas campestris* XopD acts at downstream of the activation of the MAPK cascade by inhibiting the activity of the transcription factor MYB30^31^. A wide range of *P. infestans* RXLR effectors also exhibit activities of suppressing flg22-triggered immunity^12^, indicating that oomycete RXLR effectors may share similar functions with bacterial effectors to manipulate host PTI.

CRN effectors were initially obtained from *P. infestans* and named because of their cell death-inducing activities in plants^32^. Recent studies showed that only a few CRN effectors cause cell death, whereas most of them can suppress cell death induced by PAMPs or other effectors^33^,^34^. CRN C-terminal regions contain many conserved domains that drive CRN diversity by chimeric recombination^16^,^33^. The DC domain has similarity to protein kinases and *P. infestans* CRN8 containing this domain may suppress plant defense and cause cell death^16^,^35^. *P. sojae* CRN108 has a helix-hairpin-helix motif and suppresses expression of plant *heat shock protein* genes by targeting to their promoters^36^. Functions of other domains are almost unknown.

Previously, we identified PsCRN63 from *P. sojae* and demonstrated that it induced cell death in plants while PsCRN115 suppressed this cellular process although their functional C-terminal regions contain only four amino acids difference^37^. PsCRN115 may mediate disease resistance and abiotic stress tolerance when it was expressed in plants^38^. They manipulate plant cell H_2_O_2_ homeostasis by interaction with and effecting stability of plant catalases, the essential enzymes of scavenging reactive oxygen species (ROS)^39^.

To further understand the functions and molecular mechanisms of oomycete CRN effectors in pathogenesis, we first measured ability of PsCRN63 to suppress flg22-induced marker gene *FRK1* using transient expression in protoplasts of the distantly-related non-host plant *Arabidopsis*. Secondly, we tested whether PsCRN63 blocks PTI signaling pathway in *Arabidopsis* using the stable transgenic lines. Finally, we showed that dimerization of PsCRN63 was essential for its functions inside plant cells and that the dimerization congruously exists among *Phytophthora* CRN effectors. The results provide novel insight into the molecular mechanisms underlying how *Phytophthora* pathogens manipulate plant immunity to facilitate infection.

## Results

### PsCRN63 inhibits flg22-induced expression of *FRK1*, a PTI marker gene

**Figure 1:**
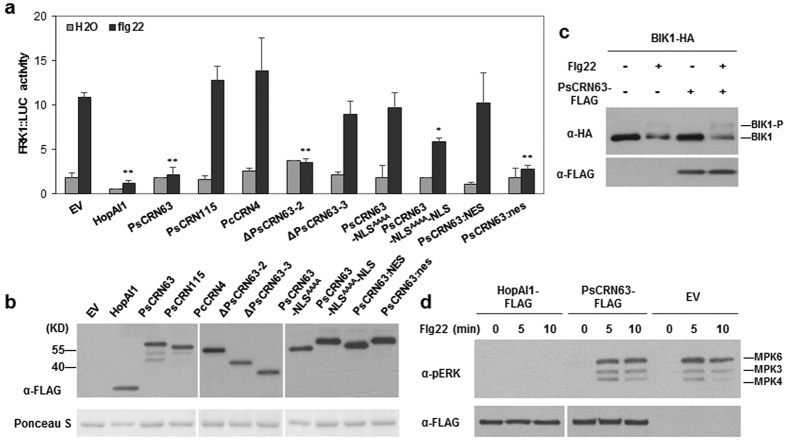
Functional analysis of PsCRN63 in Arabidopsis protoplasts. **(a**) Flg22-induced *FRK1::LUC* expression. *Arabidopsis* (Col-0) protoplasts were transfected with the indicated plasmids along with *FRK1::LUC* and *35S::RLUC*. The tested effector constructs were analyzed and displayed in Fig. S1, including HopAI1 as a positive control. *FRK1::LUC* expression activities were determined by measurements of the *LUC* reporter activity in protoplasts that were treated with H_2_O or flg22 (1 μM). Values were normalized to an internal *35S::RLUC* control. Each data point represents the mean of three replicates and error bars indicate standard deviation (*p < 0.05; **p < 0.01. Student’s *t* test). The experiments were repeated three times with similar results. **(b**) Protein expression levels determined by Western blot. Proteins coded by the constructs indicated as (**a**) were detected with an anti-FLAG antibody (upper panel) and equal loading of each sample is indicated by ponceau staining of Rubisco protein (lower panel). **(c**) Flg22-induced BIK1 phosphorylation. BIK1 phosphorylation was detected as a band-shift in an anti-HA immunoblot of total proteins prepared from the protoplasts that were transfected with/without PsCRN63 and treated with flg22 for 10 min. The result shown is representative of three independent experiments. **(d**) Flg22-induced MAPK activation. Protoplasts were transfected with HopAI1, PsCRN63 and an empty vector, and induced with flg22 at the indicated time points. Total proteins were performed by immunoblot with Phospho-p44/42 MAPK antibody. The identities of phosphorylated MAPKs in *Arabidopsis* are labeled.

To investigate whether *P. sojae* effectors interfere with the PTI, we used an *Arabidopsis* protoplast-based dual reporter assay for flg22-induced expression of *FRK1*, which is widely used as a PTI marker gene^25^,^26^. Flg22 significantly induced the expression of *FRK1* promoter-firefly luciferase reporter gene (*FRK1::LUC*) compared to H_2_O when protoplasts transfected with empty vector, which is normalized with *35S::RLUC* expression. The *P. syringae* effector HopAI1 (as a positive control) reduced *FRK1::LUC* expression by 85% upon flg22 treatment (Fig. 1a), which is consistent with the previous reports^29^. In total, three *P. sojae* RXLR effectors (Avr1k, Avr3c and Avr4/6) and four CRN effectors (CRN63, CRN115, CRN124 and CRN127) were examined, and no visible phenotype differences were observed in the protoplasts expressing the above genes. Only PsCRN63 suppressed *FRK1::LUC* induction while other six did not (Fig. 1a and Supplementary Table S1), while another cell death inducing effector PcCRN4 was used as a negatives control^40^. We therefore focused on PsCRN63 in the rest of the study.

Previously, we showed that ΔPsCRN63–2 (133–450) could induce cell death (CD) while ΔPsCRN63–3 containing aa 163–450 could not, and the activity requires nuclei localization in plant cells^37^. Here we observed that ΔPsCRN63–2, but not ΔPsCRN63–3, exhibited suppression of *FRK1* expression (Fig. 1a). Mutation of its predicted nucleus localization signal (PsCRN63-NLS^AAAA^) or C-terminal fusion with a nuclear exclusion signal (PsCRN63:NES) completely impaired its ability to inhibit flg22-induced reporter gene *FRK1* expression. Consistently, attaching with a nucleus localization signal to the C-terminus (PsCRN63-NLS^AAAA^-NLS) could partially recover the suppression activity of PsCRN63-NLS^AAAA^. PsCRN63:nes which has a nonfunctional NES (nes) fused to the C-terminus still retained its ability to inhibit *FRK1* (Fig. 1a), indicating that PsCRN63 needs to target plant cell nucleus to suppress PTI. All these tested proteins and mutants were properly expressed at the expected sizes with comparable levels in *Arabidopsis* protoplasts as indicated by Western blot analysis (Fig. 1b), indicating that loss of suppression activity is not caused by the expression levels of the proteins. These results indicated that identical requirement of PsCRN63 domains for the suppression of *FRK1* expression in *Arabidopsis* and CD-inducing activity in *Nicotiana benthimiana* and soybean.

### Lysine329 residue is essential for PsCRN63 activity

The amino acid sequences of PsCRN63 and PsCRN115 differ in only 4 residues between amino acids 133–450 (Supplementary Fig. S2a). Amino acid-swapping experiment showed that the K329E substitution completely abolished the cell death-inducing activity of PsCRN63, while the E329K substitution allowed PsCRN115 to induce cell death in *Nicotiana benthimiana* (Supplementary Fig. S2b). Similarly, PsCRN63:K329E was abolished in its ability to inhibit *FRK1::LUC* induction by flg22 whereas PsCRN115:E329K gained the PTI-suppression activity when expressed in *Arabidopsis* protoplasts (Supplementary Fig. S2c,d). These results further support that the PTI-suppression activity and cell death-inducing ability of PsCRN63 are strongly correlated.

### PsCRN63 does not affect MAPK activation and BIK1 phosphorylation

To investigate the potential mechanisms by which PsCRN63 inhibits PTI, we investigated two early biochemical events of PTI signaling pathways, flg22-induced MAPK activation and BIK1 phosphorylation^41^,^42^,^43^. The expression of PsCRN63 in protoplasts was unable to prevent phosphorylation of BIK1 (Fig. 1c) and MPK6/3/4 (Fig. 1d) after flg22 treatment. In contrast, HopAI1, the positive control, blocked MAPK activation as reported^29^. These results indicated that PsCRN63 might act downstream of the MAPK cascades in PTI signaling.

### PsCRN63 contains unknown protein modification(s) in N-terminus

**Figure 2:**
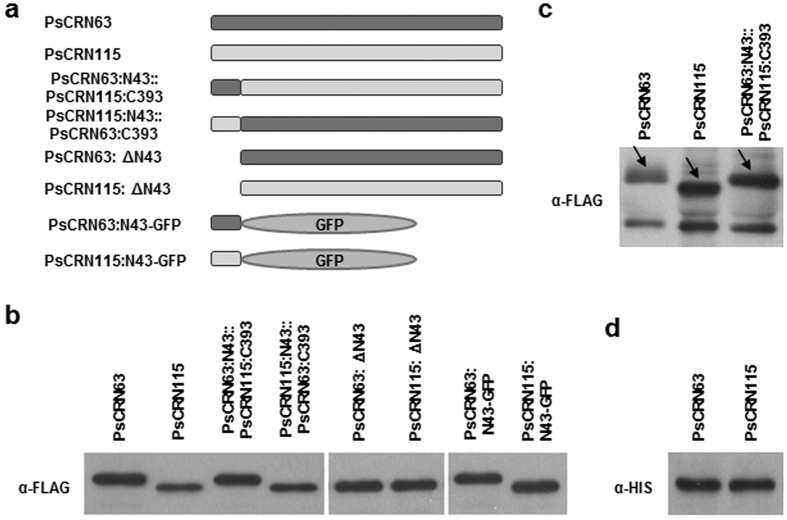
Determination of modification (s) in PsCRN63. **(a)** Schematic view of PsCRN63 and PsCRN115 along with corresponding artificial mutants. The dark grey strips represent PsCRN63, while the light grey ones symbolize PsCRN115. **(b–d**) Western blot analysis of the indicated proteins expressed in *Arabidopsis* (**b**), *N. benthimiana* (**c**) and *E. coli* **(d**). The proteins encoded by the indicated constructs were detected by immunoblot with an anti-FLAG (**b,c**) or anti-HIS (**d**) antibody. Solid arrows indicated the expected bands of proteins.

Interestingly, we noticed that PsCRN63 showed a slower migration than PsCRN115 in SDS-PAGE when expressed in *Arabidopsis* (Figs 1b and 2b) and *N. benthimana* (Fig. 2c). However, they shared the same sizes when they were produced in *E. coli* (Fig. 2d). Considering that the two CRNs have identical predicted molecular weight, we suppose that these proteins differentially modified post-translationally *in planta*.

To map site(s) required for the modification in PsCRN63, we generated domain-swapping constructs that are summarized in Fig. 2a. We found that PsCRN63:N43::PsCRN115:C393 (amino acids 15–57 of PsCRN63 fused with amino acids 58–450 of PsCRN115) but not PsCRN115:N43::PsCRN63:C393 displayed the similar migration patterns as PsCRN63 (Fig. 2b,c). In contrast, the migration of the region (aa 15–57) deletion mutant PsCRN63: ΔN43 is in accordance with PsCRN115: ΔN43 (Fig. 2b). These results indicated that the differential modification(s) depended on amino acids 15–57 of PsCRN63. Furthermore, we found that PsCRN63:N43-GFP and PsCRN115:N43-GFP also exhibited different band sizes in accord with that occurred between PsCRN63 and PsCRN115 (Fig. 2b). These results together suggest that amino acids 15–57 of PsCRN63 is necessary and sufficient to support the differential modification(s) in PsCRN63. Since the region (aa 15–57) is not required for CD-induction and PTI-suppression activities of PsCRN63, we didn’t focus on the modification(s) anymore.

### *PsCRN63*-transgenic plants are impaired in disease resistance

To further analyze the effect of PsCRN63 on plant defense, we generated *PsCRN63*-transgenic *Arabidopsis* plants using an oestrogen-inducible promoter. As shown in Supplementary Fig. S3a, we obtained 6 independent lines in which PsCRN63 accumulated at the expected bands after estradiol induction. We selected two lines (12# and 13#) for further characterization because of highly expression levels. The T2 progenies of the *PsCRN63*-transgenic plants also have a stable and high expression level of *PsCRN63* (Supplementary Fig. S3a). Generally, we found that *PsCRN63*-transgenic plants grow relatively smaller than the wild type without estradiol treatment (Supplementary Fig. S3b,c), and an exaggerated growth inhibition was found under estradiol treatment (Supplementary Fig. S3c). We owed this phenotype alteration to the fact that oestrogen-inducible promoter usually has leaking expression and PsCRN63 is toxic to plant cells although it can not trigger visible cell death in *Arabidopsis*.

**Figure 3:**
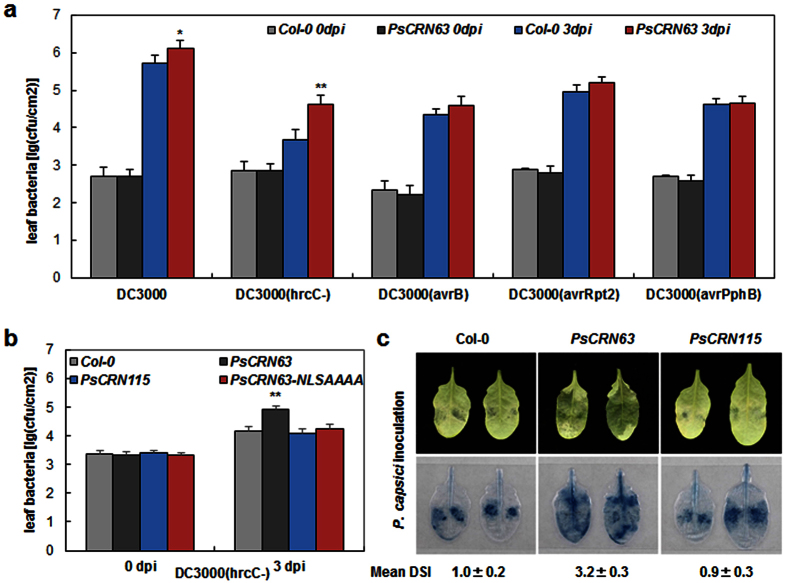
Impaired disease resistant levels in *PsCRN63*-transgenic *Arabidopsis* plants. **(a**) Bacterial population in the *Arabidopsis* leaves. *P. syringae* strains DC3000, DC3000 (*hrcC*^*−*^), DC3000 (*avrB*), DC3000 (*avrRpt2*) and DC3000 (*avrPphB*), representing the wild type of *P. syringae*, a mutant lacking of a functional type III secretion system, and three isolates carrying the indicated avirulent genes. They were infiltrated into leaves of wild-type *Arabidopsis* (Col-0) and *PsCRN63*-transgenic lines that both were pre-treated with estradiol. Bacterial population was measured at the indicated times (mean ± s.d.; n ≥ 6; *P < 0.05; **P < 0.01; Student’s *t*-test). **(b**) Comparison of resistant levels in *PsCRN63*-, *PsCRN115*- and *PsCRN63*^*AAAA*^- transgenic plants. The indicated *Arabidopsis* lines were inoculated with *P. syringae* DC3000 (*hrcC*^*−*^) and the bacterial population was determined at the indicated times (mean ± s.d.; n ≥ 6; **P < 0.01, Student’s *t*-test). **(c**) Aggravated lesions of *P. capsici* on *PsCRN63*-transgenic plants. *P. capsici* zoospore suspensions were used to inoculation on leaves pre-treated with estradiol and the photographs were taken at 36 hpi (upper panel. The lower panel shows the typical phenotypes under trypan blue staining and disease severity index (DSI) were labeled at the bottom.

*Arabidopsis* is a susceptible host to *Pseudomonas syringae* pathovar *tomato* (*Pst*) DC3000, while the mutant strain *P. syringae* DC3000 (hrcC^−^), which carries a collection of PAMPs but lacks a functional type III secretion system, is almost nonpathogenic^44^. We inoculated *PsCRN63*-transgenic plants with *P. syringae* DC3000 and DC3000 (hrcC^−^) to test if PsCRN63 undermines plant basal resistance. As shown in Fig. 3a, *PsCRN63*-transgenic plants supported approximately 9-fold greater DC3000 (hrcC^−^) bacterial growth than did the wild type plants, on the basis of nearly the same initial bacteria population. The *Pst* DC3000 bacteria grew to a 2- to 3-fold higher population in transgenic lines compared with WT plants after 3 days inoculation (Fig. 3a).

At the same time, we generated transgenic lines that expressed *PsCRN63-NLS*^*AAAA*^ and found that *PsCRN63-NLS*^*AAAA*^-transgenic seedlings only showed partial growth inhibition phenotype compared with the wild type (Supplementary Fig. S3b). Then, we complementally inoculated *PsCRN63-NLS*^*AAAA*^ transgenic plants with DC3000 (hrcC^−^) and found that the NLS inactive mutant of PsCRN63 as well as PsCRN115 were completely unable to enhance *in planta* growth of *Pst* DC3000 (hrcC^−^) (Fig. 3b). Thus, PsCRN63 can markedly compromise PTI resistance in *Arabidopsis*, and it is likely dependent on its nucleus localization.

The *PsCRN63*-transgenic lines were also inoculated with *Pst* strains carrying effector genes *avrB*, *avrRpt2* or *avrPphB*, which elicit ETI resistance mediated by the cytoplasmic immune receptors RPM1, RPS2 and RPS5, respectively^1^. In these three cases, the *PsCRN63*-transgenic lines supported less than 2-fold increase in bacterial growth, which is similar to the WT (Fig. 3a). Thus, the *PsCRN63* transgene mostly compromised PTI, rather than affected RPM1-, RPS2- and RPS5-dependent ETI.

To evaluate the virulence of PsCRN63 on plant resistance to oomycete pathogens, we inoculated the transgenic plants with a compatible oomycete pathogen *P. capsici.* Compared to the wild type, the expression of *PsCRN63* tremendously enhanced colonization of *Arabidopsis* by *P. capsici* 36 hpi, in which PsCRN63 caused about ~3.2 fold increase of the lesion size (Fig. 3c). Nevertheless, *PsCRN115*-trangenic plants showed similar levels of lesion size compared to Col-0. Taken together, these results suggest that expression of *PsCRN63* weakened plant resistance to pathogens.

### PsCRN63 suppresses callose deposition and affects expression of defense-related genes

**Figure 4:**
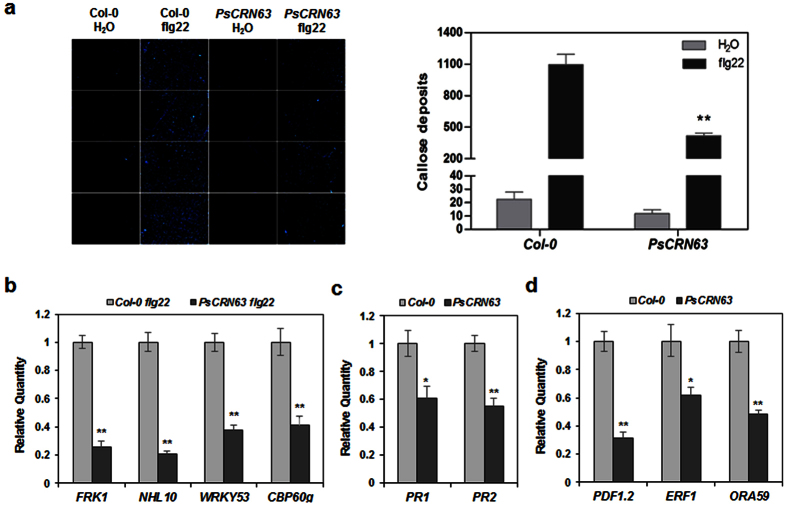
Suppression of callose deposition and expression of defense-related genes by PsCRN63. **(a**) Diminished callose deposition in *PsCRN63*-transgenic plants. Indicated *Arabidopsis* lines were infiltrated with H_2_O or flg22, and callose deposits were photographed at 8 hpi. The figure shows representative images. The quantitation of callose deposits was labeled on the right. Each data point represents the mean of six replicates. Error bars indicate standard deviation (**p < 0.01, Student’s *t* test). **(b)** Transcriptional levels of the PTI marker genes under flg22 treatment. Real-time RT-PCR were carried out to analyze expressional levels of the genes in *Arabidopsis*. *AtACT1* was used as a reference gene. Each data represents the mean of three replicates. Error bars indicate standard deviation (**p < 0.01, Student’s *t* test). The experiments had three biological repeats with similar results. **(c,d)** Inspection of transcriptional levels of the defense-related genes. The data was recorded and calculated with same methods as described above.

To further explore the mechanisms of PTI-suppression function of PsCRN63, *PsCRN63*-transgenic lines were firstly tested for callose deposition in response to flg22. We found that the expression of *PsCRN63* in transgenic plants suppressed flg22-induced callose deposition to 30%–40% of that in wild type (Fig. 4a). Besides, four widely-used PTI marker genes were also tested in *PsCRN63*-transgenic plants^45^. As shown in Fig. 4b, the levels of *FRK1, NHL10*, *WRKY53*, and *CBP60g* transcripts were reduced to ~30%, ~20%, ~40%, ~50% of that in WT plants after flg22 treatment, respectively (Fig. 4b). Together, these results indicate that PsCRN63 suppresses PTI response including callose deposition.

To further investigate the role of PsCRN63 in impairing basal resistance, we measured transcription levels of the defense-related genes in *PsCRN63*-transgenic *Arabidopsis* by qRT-PCR. As shown in Fig. 4c, two salicylic acid (SA) signal-induced and antimicrobial *PR* genes, *PR1* and *PR2*^46^, were down-regulated to nearly 55% compared with WT plants (Fig. 4c). Futhermore, we selected three marker genes involved in jasmonic acid (JA)/ethylene (ET)-mediated defense pathway^47^. Generally, we found that presence of PsCRN63 was able to cause nearly 2-fold decrease of *ERF1*, *ORA59* in transcription level, meanwhile inhibit *PDF1.2* approximately 3-fold decrease of transcripts (Fig. 4d).

### PsCRN63 can form a homo-dimer via an inverted association manner

**Figure 5:**
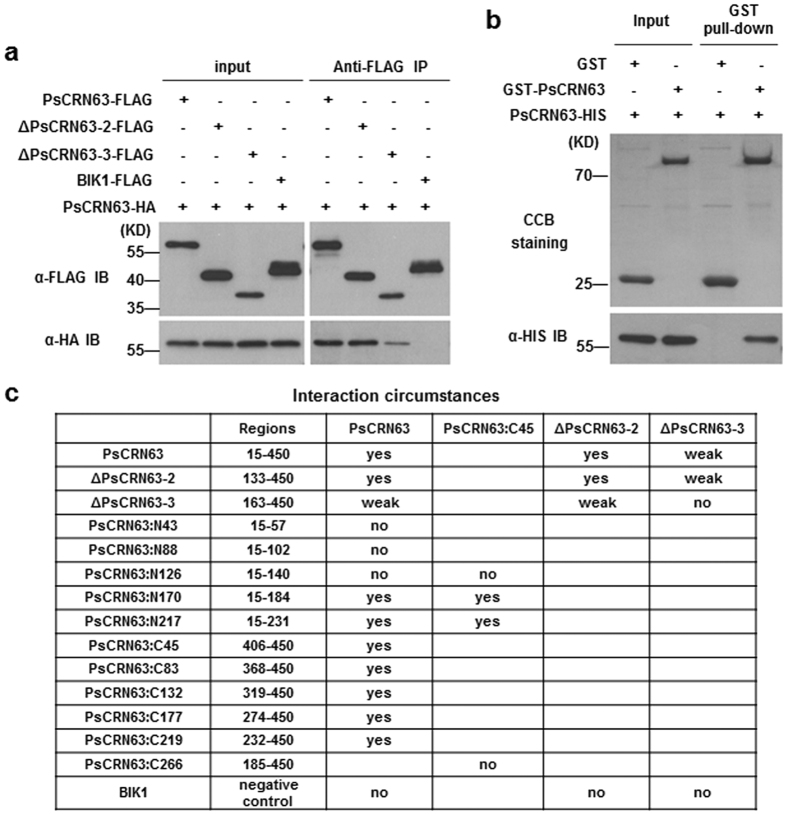
Dimerization of PsCRN63 via an inverted association manner. **(a)** Dimerization of PsCRN63 *in vivo*. Indicated plasmids combination were co-expressed in WT *Arabidopsis* protoplasts, extracted total protein was immunoprecipitated with anti-FLAG antibody (α-FLAG IP), and the bound protein was detected by immunoblot with the indicated antibodies. **(b**) Dimerization of PsCRN63 *in vitro*. A His-tagged PsCRN63-HIS and a GST-tagged GST-PsCRN63 or GST recombinant proteins were affinity purified, and the protein-protein interaction was tested by a GST pull-down assay. The amounts of bound protein PsCRN63-HIS was analyzed by anti-His immunoblot (IB) and the gel was stained with Coomassie Brilliant Blue (CBB) to show amounts of the indicated GST-tagged proteins, which termed CBB staining. **(c**) A complete summary of different sections involved in dimerization of PsCRN63. The initialization-termination sites of truncated mutants were shown in the column “Regions”. All the mutant constructs in the table were exhibited in Fig. S1 and “yes” represents that there is a protein-protein interaction but “no” means no interaction. Besides, “weak” indicates the less amount of the protein association.

It has been shown that some effectors form dimers *in planta*, such as CRN8 from *Phytophthora infestans*^35^. We implemented the co-immunoprecipitation (co-IP) assay in *Arabidopsis* protoplasts to determine whether PsCRN63 can form a dimer. As shown in Fig. 5a, PsCRN63-FLAG, but not BIK1-FLAG, specifically interacted with PsCRN63-HA (Fig. 5a). Meanwhile, ΔPsCRN63–2-FLAG (133–450) also interacted with PsCRN63-HA, whereas interaction between ΔPsCRN63–3-FLAG (163–450) and PsCRN63-HA was weakly detected (Fig. 5a,c). Glutathione S-transferase (GST) pull-down assay also showed that a GST-tagged PsCRN63 was able to bind a His-tagged PsCRN63, indicating that PsCRN63 can form a homo-dimer by direct inter-molecular interaction *in vitro* (Fig. 5b). These results suggest that PsCRN63 associates *in vivo* and *in vitro* in a specific manner and the association may be related to its cell death-inducing and PTI-suppression activities.

To determine the precise subsections of PsCRN63 that dominate the formation of homo-dimer, we designed a series of progressive truncated PsCRN63 mutants to fuse with a GFP-HA in the C-terminus (Supplementary Fig. S1) and tested their interactions. As shown in Fig. 5c, successive deletion up to residues at 140 from C-terminus (PsCRN63:N126) abolished their interaction with PsCRN63-FLAG whereas deletion up to residues at 184 (PsCRN63:N170) retained interaction activities (Fig. 5c and Supplementary Fig. S4a). The results suggest that N-terminal segment (141–184) of PsCRN63 is critical to form a homologous complex. At the same time, deletion up to residues at 406 from N-terminus (PsCRN63:C45) did not affect interaction, suggesting that C-terminal segment (406–450) is also essential (Fig. 5c and Supplementary Fig. S4b). Next, we demonstrated that PsCRN63:N126 or PsCRN63:C266 could not interact with PsCRN63:C45, but PsCRN63:N170 as well as PsCRN63:N217 could associate with PsCRN63:C45 (Fig. 5c and Supplementary Fig. S4c). Since the N-terminal segment of PsCRN63 specially combines the C-terminal segment, we infer that PsCRN63 can form a homo-dimer through an inverted association manner (Supplementary Fig. S4d).

### Similar inverted association manner exists among *Phytophthora* CRN effectors

Chimeric recombination drives CRN diversity^16^. Next, we examined whether the N-terminal and C-terminal segments that mediate protein interaction were conserved in other CRN effectors. Using BLAST analysis against *P. sojae, P. ramorum, P. infestans* and *P. capsici* genome sequences with these two segments as queries, we obtained 32 *Phytophthora* effectors. As shown in Supplementary Fig. S5, all these CRN homologs contain at least one of the conserved N-terminal and C-terminal domains with high similarity (Supplementary Fig. S5).

**Figure 6:**
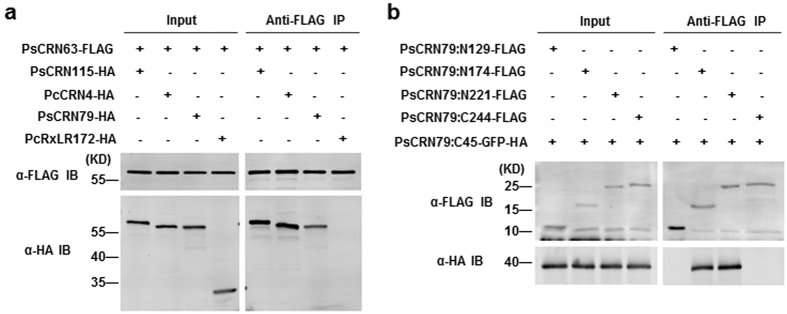
Interactions among CRN family members via conserved N-terminal and C-terminal domains. **(a)** Hetero-dimerization among *phytophthora* CRN effectors. PsCRN115-HA, PcCRN4-HA, PsCRN79-HA and PcRxLR172-HA were expressed in WT *Arabidopsis* protoplasts, accompanied by PsCRN63-FLAG. Total protein was extracted and an α-FLAG IP experiment was conducted as described before. **(b)** Association between N-terminal segment (aa 130-174) and C-terminal segment (aa 374-418) of PsCRN79. PsCRN79:C45-GFP–HA was expressed in WT *Arabidopsis* protoplasts, along with indicated FLAG-tagged PsCRN79 derivatives, immunoprecipitated with anti-FLAG antibody, and protein–protein interaction was analyzed by immunoblot with the indicated antibodies.

We speculated that the conserved domains might mediate hetero-dimerization of these identified CRN effectors and tested the hypothesis using anti-FLAG co-IP in *Arabidopsis* protoplasts. As shown in Fig. 6a, three representative effectors, PsCRN115, PsCRN79 and even a *P. capsici* effector CRN4 can interact with PsCRN63 (Fig. 6a). In contrast, PcRxLR172-HA (as a negative control, lacking of N-terminal or C-terminal domains) can not interact with PsCRN63 (Fig. 6a). The results suggest that the conserved N-terminal and C-terminal domains may facilitate dimerization of the effectors.

To confirm the above observations, we constructed the truncated mutants of PsCRN79 and implemented the co-immunoprecipitation (co-IP) assay in *Arabidopsis* protoplasts. We found that PsCRN79:N129 or PsCRN79:C244 could not interact with PsCRN79:C45 while PsCRN79:N174 and PsCRN79:N221 could (Fig. 6b). This results suggest that PsCRN79, similar to PsCRN63, also associates *in planta* in a specific manner and the conserved domains determine an inverted interaction.

### Dimerization is positively correlated with PTI-suppression activity of PsCRN63

**Figure 7:**
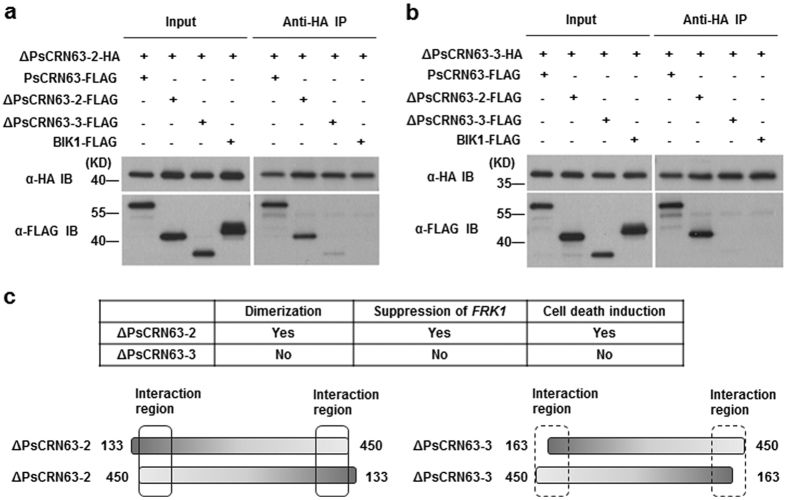
Dimerization of PsCRN63 is associated with PTI suppression and cell death induction. **(a,b)** Homo-/hetero-dimerization of PsCRN63, ΔPsCRN63-2 and ΔPsCRN63-3 *in planta*. BIK1-FLAG, PsCRN63-FLAG, ΔPsCRN63-2-FLAG and ΔPsCRN63-3-FLAG were transfected into Col-0 protoplasts, accompanied by ΔPsCRN63-2–HA (**a**) or ΔPsCRN63-3-HA (**b**), respectively. Total protein was immunoprecipitated with anti-HA antibody (α-HA IP) and protein–protein interaction was analyzed by Anti-HA immunoblot. **(c)** Dimerization of PsCRN63 is required for PTI inhibition and cell death induction. The left two strips represent ΔPsCRN63-2 and ΔPsCRN63-3 is in the right side. Solid elliptical rectangle means where interaction occurs, while dashed elliptical rectangle symbolizes no interaction.

We performed the anti-HA co-IP in *Arabidopsis* protoplasts and found ΔPsCRN63–2-HA strongly interacts with ΔPsCRN63–2-FLAG but was slightly capable of interacting with ΔPsCRN63–3-FLAG (Fig. 7a). Nevertheless, ΔPsCRN63–3-HA was completely unable to interact with ΔPsCRN63–3-FLAG (Fig. 7b). Thus, we may conclude that ΔPsCRN63–2 rather than ΔPsCRN63–3 facilitates dimerization, and probably because of the deficiency of the N-terminal domain (aa 141–184) in ΔPsCRN63–3 (Fig. 7c). This results may explain the observation about different activities of ΔPsCRN63–2 and ΔPsCRN63–3 in PTI signaling inhibition and cell death induction (Fig. 7c).

## Discussion

In this study, we used a protoplast-based reporter assay in *Arabidopsis* to assess the potential for several CRN effectors from *P. sojae* to suppress PTI using flg22-induced expression of the PTI marker gene *FRK1* as a reporter^25^,^26^. We found PsCRN63 was able to suppress flg22-mediated induction of pFRK1-Luc activity in non-host plant *Arabidopsis*, and this suppression function is correlated with its CD-inducing activity. We further demonstrated PsCRN63 suppressed PTI response including callose deposition in *Arabidopsis* using the stable *PsCRN63*-transgenic lines, and the transgenic plants are impaired in disease resistance to the bacterial pathogen *P. syringae* DC3000 as well as the oomycete pathogen *P. capsici.* Furthermore, our study revealed that the conserved N-terminal along with the C-terminal domains from PsCRN63 facilitates dimerization through an inverted association manner. In addition, this novel association of PsCRN63 correlates with its activities of CD-induction and PTI-suppression.

The initiation of PTI signaling in plants following PAMP perception engages a multitude of processes, including PRR activation^24^, MAP kinase signaling cascades^48^ and transcriptional reprogramming^49^. *FRK1* is a rapidly up-regulated gene and has been widely used as a reporter gene of flg22-dependent activation^12^,^25^. Many phytopathogen effectors appear to suppress flg22-triggered immunity by acting at different stages of PTI signaling pathway. For instance, *P. syringae* effectors AvrPto and AvrPtoB block FLS2-mediated signaling transduction in *Arabidopsis*^26^,^27^, *P. syringae* effector HopAI1 inhibits MPK4 kinase activity to block MAPK cascades^50^ and a set of RXLR effectors from *Phytophthora infestans* manipulate early stages of flg22-triggered signaling^12^. In our study, PsCRN63 notably suppresses *FRK1::LUC* induction that is comparable to the HopAI1 in the non-host *Arabidopsis*. However, two early biochemical events of PTI signaling pathways, flg22-induced MAPK activation and BIK1 phosphorylation were intact upon *PsCRN63* expression in protoplasts. These results indicated that PsCRN63 might act downstream of MAPK cascades in PTI signaling. Additional experiments are required to determine which steps PsCRN63 interferes with, which may account for the suppression of PsCRN63 on expression of *FRK1* in *Arabidopsis*.

Some effector proteins of plant pathogens are known to have dual activities during infection. On the one hand, an effector can trigger a rapid HR or HR-like response when the corresponding R protein is present. On the other hand, the effector may suppress PTI or ETI and thereby enhance pathogenesis in the susceptible host cells^51^. In previous study, we showed that PsCRN63 triggered cell death in *N. benthamiana* and host soybean^37^. In this study, we generated stable *PsCRN63*-transgenic *Arabidopsis* plants whose expression is driven by oestrogen-induced promoter. It is interesting that over-expression of *PsCRN63* leads to invisible phenotype in neither *Arabidopsis* plants nor *Arabidopsis* protoplasts, mainly because functional differentiation of PsCRN63 on multiple hosts. The *PsCRN63*-transgenic plants showed a remarkable suppression of flg22-induced callose deposition and expression of the widely-used PTI marker genes. *P. sojae* does not possess flagellin, but contains many known PAMPs^5^,^6^,^7^. The pathogen may produce PsCRN63 to target conserved PTI signaling pathway to promote infection.

In addition, the *PsCRN63*-transgenic *Arabidopsis* showed enhanced susceptibility to the virulent isolate *P. syringae* DC3000 as well as compatible oomycete pathogen *P. capsici*. This is consistent with our previous results that expression of PsCRN63 in planta enhanced the susceptibility of *N. benthamiana* to P. capsici infection^39^. It is also worth mentioning that *PsCRN63*-transgenic plants supported approximately 9-fold greater DC3000 (hrcC^−^) bacterial growth than did the wild type plants rather than DC3000 strains carrying effector genes *avrB*, *avrRpt2* or *avrPphB,* since the former strain is commonly regarded as a PMAP complex for lacking of type-three secretion system (TTSS). We speculate that PsCRN63 enhance growth of *P. syringae* DC3000 (hrcC^−^) via intense PTI suppression, our experimental data clearly demonstrated that PsCRN63 impairs resistance and facilitates infection by suppressing plant innate immunity in host cells.

Plant pathogen effectors either function in the apoplast (extracellular effectors) or traffic into the host cell (intracellular effectors) where they modulate host signaling pathways to benefit the pathogens. Among intracellular effectors, a wide range of effector proteins have been identified to target the nucleus or proteins function in this compartment. For example, *Xanthomonas campestris* pv. *vesicatoria* effector AvrBs3 was shown to localize to the plant cell nucleus and mimic eukaryotic transcription factors, causing hypertrophy of plant mesophyll cells^52^. Another type III secretion effector from *Xanthomonas euvesicatoria* (Xcv) XopD, was shown to manipulate host nuclear sumoylation status and repress ethylene-induced transcription^53^. In previously study, PsCRN63 was predicted to contain a Nuclear Localization Signal (NLS), which is required for PsCRN63 to induce cell death in *N. benthamiana*^37^. In this study, we found that localizing to the nucleus of PsCRN63 is also essential for suppression of flg22-induced *FRK1* expression. Meanwhile, the NLS inactive mutant of PsCRN63 were completely unable to enhance *in planta* growth of *Pst* DC3000 (hrcC^−^).

Post-translational *in vivo* modifications are significant variations to the cellular protein component, and many protein modifications including phosphorylation, ubiquitination and sumoylation are thought to be implicated in plant immunity^54^. As reported, the type III effector HopU1 is a mono-ADP-ribosyltransferase, and it suppresses plant innate immunity in a manner dependent on its ADP-ribosylation of GRP7^55^. Furthermore, *Xanthomonas campestris* pathovar *campestris* type III effector AvrAC enhances virulence and inhibits plant immunity by specifically targeting BIK1 and RIPK, by means of adding uridine 5′-monophosphate to these two receptor-like cytoplasmic kinases^43^. Since host proteins suffering from modification received most attention in recent years, very few work reported modified proteins from plant pathogens. In this study, we observed PsCRN63 showed a slower migration than PsCRN115 in SDS-PAGE, and we suppose that PsCRN63 might have unknown modifications *in planta.* Subsequent results revealed that the amino acids 15–57 of PsCRN63 is necessary and sufficient to support the modifications in PsCRN63, however, the N-terminal region (aa 15–57) seems not required for its virulence activity. We had implemented mass spectrometry of PsCRN63 recombinant protein (expressed alone in *N. benthimana* or in *Arabidopsis,* respectively), yet unfortunately we got little valuable information to uncover its modifications. The role of the modifications in PsCRN63 is still unclear and needs to be further elucidated in future.

We discovered that PsCRN63 form *P. sojae* can form a homo-dimer *in vivo* and *in vitro,* and this association occurs in a specific manner. Unfortunately, the conserved motifs of CRN effectors, such as FLAK and HVLVVVP, were not involved in this association. Significantly, the association of PsCRN63 was demonstrated to be related to its activities of CD-induction and PTI-suppression. This reminds us of another effector CRN8 from *Phytophthora infestans,* which forms a dimer or multimer in *N. benthamiana*. Similarly, CRN8 also localizes to the host nucleus and the localization is required for triggering cell death^35^. Laborious interaction assessment of different truncated mutants derived from PsCRN63 demonstrated that PsCRN63 can form a homo-dimer through an inverted association manner, on the basis of the N-terminal segment of PsCRN63 specially combines its C-terminal segment. Recent study showed that PsCRN63 interacts with plant catalases to regulate plant cell death^39^. However, the relationship between dimerization and the interaction with catalases is still unknown.

We found that at least 32 *Phytophthora* effectors shared high sequence similarity with PsCRN63 in either N-terminal or C-terminal domains that determine an inverted combination. As indeed, several other CRN effectors containing the domains could interact with PsCRN63 to form hetero-dimers and PsCRN79 also exhibited self-association. According to the observation that dimerization is crucial for virulent functions of PsCRN63, we have no reason for doubting various homo-/hetero-interactions among these effectors should have impact on intracellular processes especially plant immunity in hosts. Recent study showed that PsCRN115 can suppress CD induced by PsCRN63^37^, whether this suppression requires hetero- dimerization is of concern and needs further investigation. Also, we suggest that dimerization is a necessary but not sufficient condition for CD-induction and PTI-suppression of PsCRN63, considering PsCRN115 can form a homo-dimer as well. Although several effectors selected for dimerization detection failed to suppress flg22-mediated induction of pFRK1-Luc activity in protoplasts (data not shown), we thought the subtle mechanism under this “calm” surface should be identified and need further investigation. These results exhibit a spectacular view that some *phytophthora* effectors function either alone or in conjunction with others to form molecular complex to disturb signal transmission and cellular processes required for immunity in *planta*. This study will advance our understanding of how oomycete effectors manipulate plant immunity to promote infection.

## Methods

### Plant Materials and Growth

*Arabidopsis thaliana* plants including WT (Col-0) were grown in a growth room at 23 °C and 70% relative humidity with a 10/14 h day/night light cycle for 5 weeks before protoplast isolation or bacterial inoculation. Alternatively, seedlings were grown on vertical phytoagar plates containing 1/2 Murashige Skoog (MS) medium, 1.5% sucrose, and 50 μM estradiol (pH5.7) in the dark or under continuous light. For *Nicotiana benthamiana*, plants were grown and maintained throughout the experiments in a growth room with an ambient temperature of 22 °C to 25 °C under a 16/8 h day/night photoperiod.

### DNA Constructs

To generate constructs for protoplast transfection assay, *P. sojae* effector genes and their derivatives were PCR-amplified and inserted between *Xho* I and *BstB* I sites of pUC19-35S-Flag-RBS vector^25^ to generate PsCRN63-FLAG, PsCRN115-FLAG, PsCRN127-FLAG, ΔPsCRN63-2-FLAG, ΔPsCRN63-3-FLAG and other constructs used in protoplast transfection. Also, *P. sojae* effector genes and their derivatives were cloned into *Kpn* I and *Sal* I sites of pUC19-35S-HA-RBS^25^ to generate PsCRN63-HA, PsCRN79-HA, ΔPsCRN63-2-HA, ΔPsCRN63-3-HA and other constructs used. Both BIK1–FLAG and BIK1–HA constructs were described previously^41^. The primers used for DNA amplification and plasmids construction of different genes are listed in Table S2.

The *PsCRN63-FLAG* fragment was excised from the pUC19-35S-PsCRN63-Flag-RBS plasmid with *Xho* I and *Spe* I then mobilized to PENTR/D-TOPO vector (Invitrogen), and subsequently recombined into the Gateway compatible pFAST-G01, which contains a GFP marker specifically expressed in seed coat to facilitate selection of transgenic seeds^56^. The resulting plasmid pFAST-pER8-PsCRN63-FLAG was used for plant transformation.

### *Arabidopsis* Protoplast Preparation and Transfection, Dual Reporter Activity Assay

Protoplast preparation and transfection were essentially as described^25^, except that the transfected protoplasts were incubated in W5 medium (154 mM NaCl, 125 mM CaCl_2_, 5 mM KCl, and 2 mM MES pH 5.7) instead of 0.4 M mannitol. *PsCRN63* and its truncation mutants were co-transfected with FRK1::LUC (firefly luciferase) and 35S::RLUC (Renilla luciferase) into *Arabidopsis* protoplasts. The protoplasts were incubated overnight under low light, treated with 1 μM flg22 (Sigma) for 3 h. Protein was then isolated, and LUC activity was measured by using the Dual-Luciferase Reporter system (Promega) according to the manufacturer’s instructions.

### MAPKs Activity Assay, BIK1 Phosphorylation and Migration Shift Assay

Protoplasts were isolated and transfected with PsCRN63-FLAG, HopAI1-FLAG or empty vector as described before^25^. The transfected protoplasts were treated with water or 1 μM flg22 for 0, 5, 10 min before protein isolating for immunoblot analyses. The protein concentration was determined using a Bio-Rad Bradford protein assay kit, and equal amounts of total protein were electrophoresed on 10% SDS–PAGE. An anti-pERK antibody (no. 4370S, Cell Signaling) was used to determine phosphorylation state of MPK3, MPK4 and MPK6 in an immunoblot.

*Arabidopsis* protoplasts were transfected with HA-tagged BIK1 alone, or together with PsCRN63-FLAG, and then treated with 1 μM flg22. Total protein was extracted at 10 min. Samples were separated by 10% SDS–PAGE gels followed by anti-HA immunoblot.

### Agrobacterium tumefaciens Infiltration Assay

The *A. tumefaciens* strain GV3101 in our lab was used for this experiment^37^. For infiltration, recombinant strains were cultured at 28 °C, 220 rpm for 48 h until reaching appropriate concentration. The cells were collected by centrifugation (3,000 g, 5 min), followed by washed three times in 10 mM MgCl_2_, and then resuspended in 10 mM MgCl_2_ to an optical density at 600 nm of 0.4 to 0.6. Infiltration experiments were performed on 7- to 8-week-old *Nicotiana benthamiana* plants. Symptom development was monitored from 4 to 7 d after infiltration, and photographs were taken after 5 d.

### Transgenic *Arabidopsis* and Inoculation Assay

To generate *PsCRN63*-transgenic plants, the PsCRN63 coding region was PCR-amplified from *Phytophthora sojae* genomic DNA, ligated into a modified pER8 binary vector^57^. The resulting clone containing *PsCRN63-FLAG* drived by the oestrogen-inducible promoter was transformed into *Arabidopsis* (Col-0) according to standard protocols. Two independent transgenic lines were selected for experiments. The transgenic plants were sprayed with 50 μM estradiol in a 0.01% silwet L-77 solution for 24 h to induce PsCRN63 protein expression.

Five-week-old plants pre-induced with estradiol for 24 hours were infiltrated with the indicated *P. syringae* bacteria at 10^6^ cfu.ml^−1^. Leaf bacterial number was determined at the indicated times after bacterial inoculation. Each data point consists of at least six replicates.

The *P. capsici* strain Pc35 used in the study were routinely cultured at 25 °C in the dark on 10% (v/v) V8 juice agar plates^36^. Then incubated mycelium in 10% (v/v) V8 broth at 25 °C for 2 days, and washed three times with sterilized water. Numerous sporangia developed after 12 h. To release zoospores, the cultures were treated in cold sterilized water for 0.5 h followed by incubation at 25 °C for 1 h. Infection assays were performed using droplet inoculations of zoospore solutions of the *P. capsici* isolate (5 μL of a 50,000 zoospores per mL solution) on detached *Arabidopsis* leaves. Disease development on *Arabidopsis* leaves was evaluated using a disease severity index as described^58^ on a scale of 0–4.

### Callose Deposition Assay

Five-week-old *Arabidopsis* leaves were infiltrated with 1 μM flg22 and collected 8 h later, then stained with aniline blue, and visualized with a fluorescence microscope as described^59^. Callose deposits were calculated using Image J software (http://www.uhnresearch.ca/wcif). Each data point consists of six replicates.

### Real-Time RT-PCR Analysis

Four-week-old seedlings after treatment with 50 μM estradiol in a 0.01% silwet L-77 solution for 24 h were infiltrated with H_2_O or 1 μM flg22, harvested after 8 h, and total RNA was extracted using TRIzol (Invitrogen) according to the manufacturer’s instruction. *Arabidopsis* cDNA was synthesized with the SuperScriptIII First-Strand Kit (Invitrogen). Real-time PCR was performed by using SYBR Premix Ex Taq^TM^ on Agilent Mx3005P real-time PCR machine, according to the manufacturer’s instructions. *ACT1* was used as a control in qRT-PCR. The primers used for qRT-PCR amplification of different genes are listed in Table S2.

### Domain Prediction and Phylogenetic Tree Analysis

Signal peptide was predicted by SignalP4.1. Domain organization and function of each proteins were predicted on the SMART and Pfam Websites. The phylogenetic tree of CRN proteins was constructed using MEGA 5.1 by the neighbor joining method and 1,000 replicates for bootstrap analysis.

### Co-Immunoprecipitation Assay

The protoplasts were transfected with the indicated constructs, incubated for 12 h, and total protein was isolated with an extraction buffer containing 50 mM HEPES-KOH (pH 7.5), 150 mM KCl, 1 mM EDTA, 0.3% Triton-X 100, 1 mM DTT, complete protease inhibitors (Roche). For anti-FLAG immunoprecipitation, total protein was incubated with an agarose-conjugated anti-Flag antibody (Sigma-Aldrich) for 4 h, washed six times with a wash buffer containing 50 mM HEPES-KOH (pH 7.5), 150 mM KCl, 1 mM EDTA, 0.3% Triton-X 100, 1 mM DTT, and the bound protein was eluted with an elution buffer (extraction buffer adding 0.5 mg ml^−1^ 3 × FLAG peptide). For anti-HA immunoprecipitation, total protein was pre-cleared with protein A agarose (Millipore) for 1 h, followed by precipitation with 2 μg anti-HA antibody (TianGen) together with protein A agarose for 4 h. Total protein and Immunoprecipitates were separated by 10% SDS–PAGE gels, and detected by immunoblot with a monoclonal anti-FLAG antibody (Sigma-Aldrich) and anti-HA antibody (Tiangen), respectively. Approximately 1% of input and a quarter of eluted protein complex were analyzed by immunoblot.

### GST Pull-Down Assay

The recombinant proteins isolated from *E. coli* were affinity purified following the manufacturer’s instruction. For GST pull-down assay, 5 μg PsCRN63-HIS and 10 μg each GST, GST-PsCRN63 were incubated on a rotator with 30 μl glutathione agarose beads (GE Healthcare) in a buffer containing 25 mM Tris-HCl (pH 7.5), 100 mM NaCl and 1 mM DTT for 2 h, then washed five times with a buffer containing 25 mM Tris-Hcl (pH 7.5), 100 mM NaCl, 1 mM DTT and 0.1% Trition-X 100. The bound protein was eluted with 15 mM GSH and His-PsCRN63 was detected by anti-His (TianGen) immunoblot.

## Additional Information

**How to cite this article**: Li, Q. *et al.* A *Phytophthora sojae* effector PsCRN63 forms homo-/hetero-dimers to suppress plant immunity via an inverted association manner. *Sci. Rep.* **6**, 26951; doi: 10.1038/srep26951 (2016).

## Supplementary Material

Supplementary Information
