# Supplementary material for: A Phytophthora sojae effector PsCRN63 forms homo-/hetero-dimers to suppress plant immunity via an inverted association manner
Source: Sci Rep. 2016 May 31;6:26951. doi: 10.1038/srep26951 (PMC4886637; doi:10.1038/srep26951)
Supplement: Supplementary Information [file srep26951-s1.pdf]

A *Phytophthora sojae* effector PsCRN63 forms homo-/hetero-dimers to suppress plant immunity via an inverted association manner

Qi Li,[1,2] Meixiang Zhang,[1] Danyu Shen,[1] Tingli Liu,[1] Yanyu Chen,[1] Jianmin Zhou,[2,*] Daolong Dou,[1,*]

[1] Department of Plant Pathology, Nanjing Agricultural University, Nanjing 210095, China

[2] Center for Genome Biology and State Key Laboratory of Plant Genomics, Institute of Genetics and Developmental Biology, Chinese Academy of Sciences, Beijing 100101, China

[*] Corresponding author: Jian-Min Zhou, Daolong Dou

Email: jmzhou@genetics.ac.cn, ddou@njau.edu.cn

Tel: +86-10-64806330, +86-25-84396973

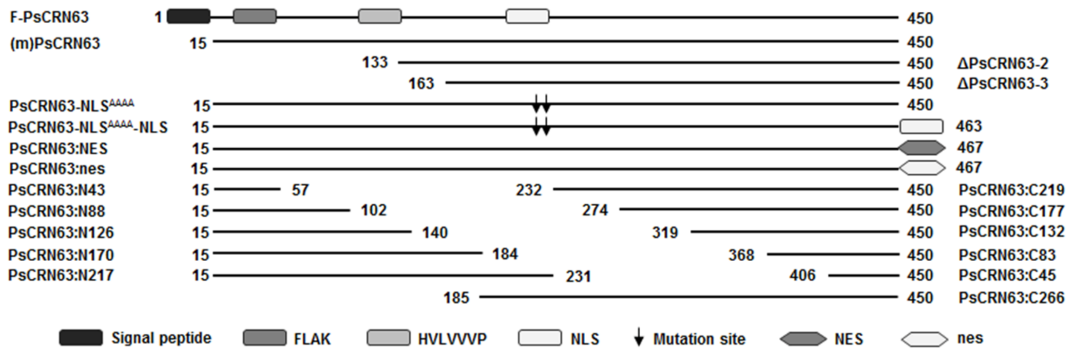

Supplementary Figure S1. Schematic view of a succession of artificial mutant constructs derived from PsCRN63.

PsCRN63 carries a N-terminal signal peptide (black rectangle), and has a conserved motif “FLAK” in its N-termini (dark grey rectangle) followed by another conserved motif “HVLVVVP” (light gray rectangle), then a nuclear localization signal (NLS) in addition (white rectangle). Little arrows represent mutation sites and flat hexagon (dark grey) symbolize a nuclear exclusion signal (NES) while the white hexagon means a nonfunctional NES (nes). The C-terminal domain carries the module with biochemical effector activity.

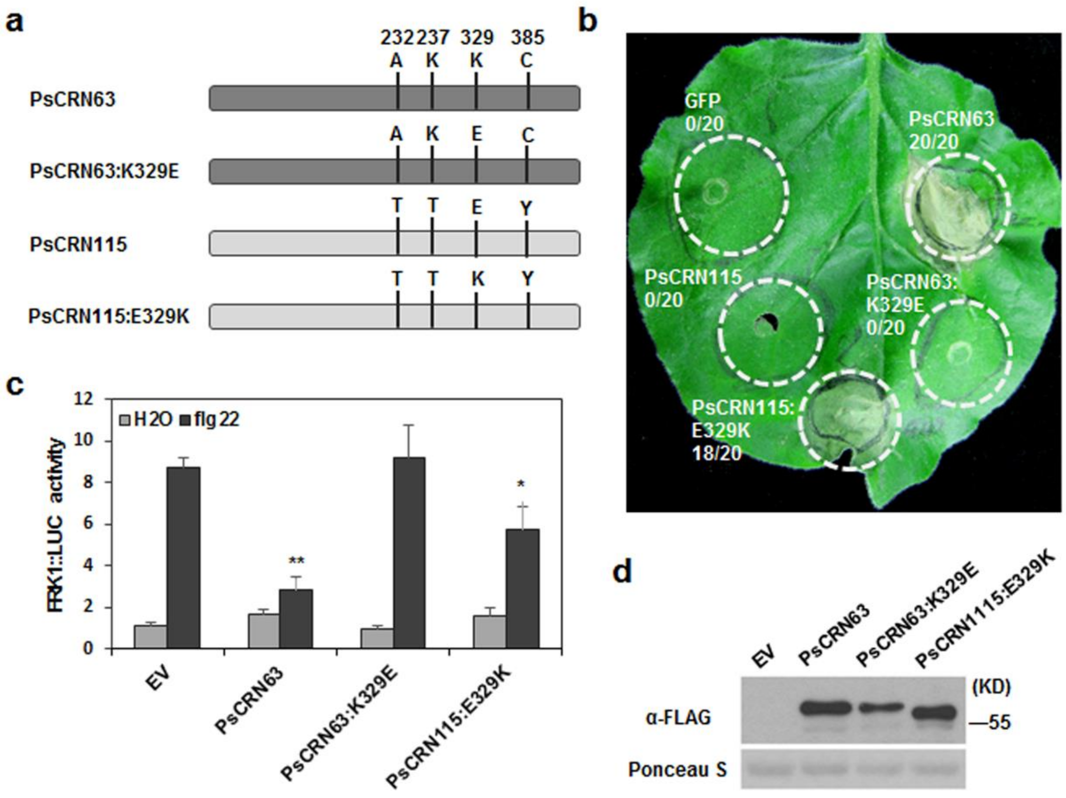

Supplementary Figure S2. PTI suppression of PsCRN63 strongly in correlation with its cell death-inducing ability.

(a) Amino acid-swapping experiment in C-terminus between PsCRN63 and PsCRN115. The upper two strips represent PsCRN63 and PsCRN63:K329E (dark grey), while the lower ones symbolize PsCRN115 and PsCRN115:E329K (light grey).

(b) Cell death induction experiments in *N. benthamiana* leaves. *N. benthamiana* was transformed with the indicated constructs or GFP (negative control) by agroinfiltration. The white circle indicates infiltration sites and the numbers show the ratio of necrotic responses to the total infiltration sites. The experiments had three biological repeats with similar results.

(c) Flg22-induced *FRK1::LUC* expression. PsCRN63, PsCRN63:K329E, PsCRN115:E329K or empty vector were transfected into Col-0 protoplasts, along with FRK1::LUC and 35S::RLUC. Samples were treated with H_2O or flg22, and the *LUC* reporter activity was determined. Each data point represents the

mean of three replicates. Error bars indicate standard deviation(*, $P < 0.05$; **, $P < 0.01$; Student's *t*-test). The experiments were repeated three times with similar results.

(d) Protein expression levels determined by Western blot. Proteins coded by the constructs indicated as (C) were detected with an anti-FLAG antibody. Equal loading is indicated by ponceau staining of Rubisco protein.

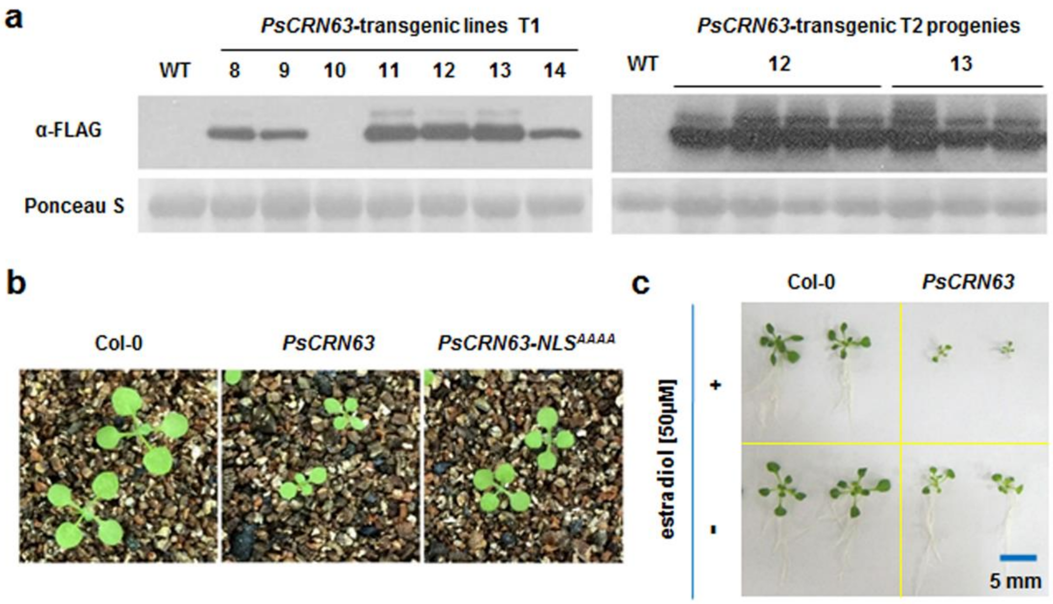

Supplementary Figure S3. Phenotype alteration of *PsCRN63*-transgenic *Arabidopsis* plants.

(a) Protein expression levels determination in *PsCRN63*-transgenic plants. Total protein isolated and detected by immunoblot with an anti-FLAG antibody. Equal loading is indicated by ponceau staining of Rubisco protein. Left panel, T1 generation of the independent *PsCRN63*-transgenic lines (8#-14#). Right panel, T2 generation of *PsCRN63*-transgenic line 12# and 13#.

(b) Phenotype of *PsCRN63* and *PsCRN63-NLS^{AAAA}* transgenic seedlings grown in roseite. Two-week-old *Arabidopsis* of the indicated genotypes under no oestrogen treatment were photographed.

(c) Phenotype of *PsCRN63* transgenic lines grown in 1/2 MS medium. Three-week-old wild type Col-0 or *PsCRN63*-transgenic *Arabidopsis* with continuous oestrogen treatment or under no oestrogen induction were photographed.

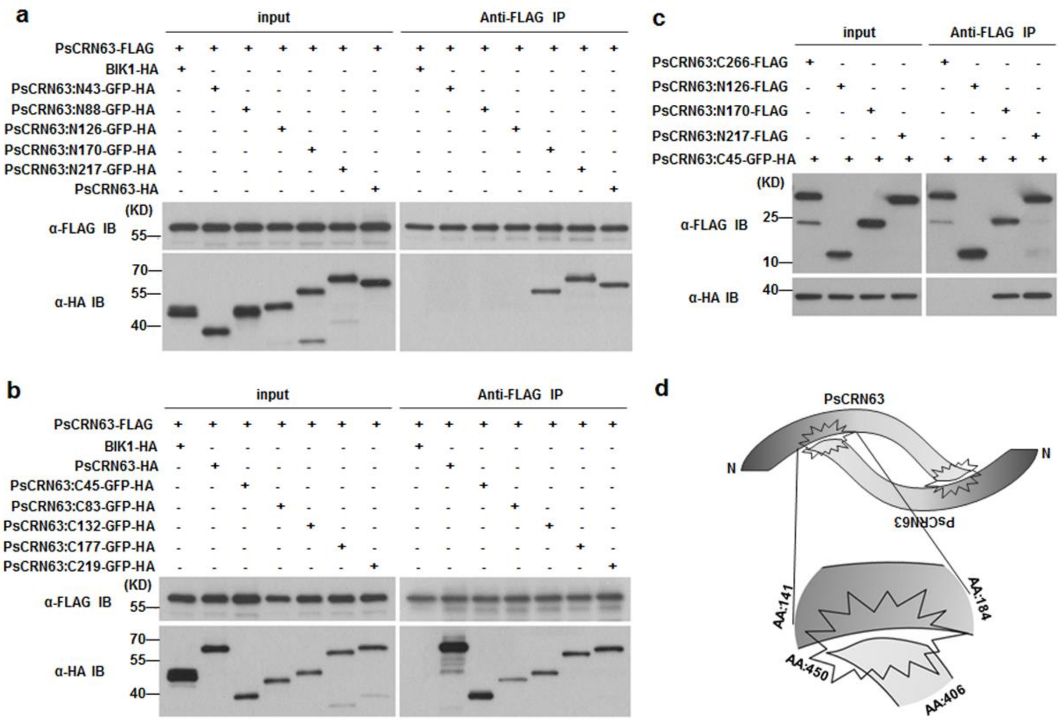

Supplementary Figure S4. Spontaneous dimerization of PsCRN63 through an inverted association manner.

(a-b) Testing essential segments of PsCRN63 for dimerization. BIK1-HA, PsCRN63-HA and its truncated derivatives were co-expressed with PsCRN63–FLAG in Col-0 protoplasts and subjected to an Anti-FLAG co-immunoprecipitation.

(c) Association between N-terminal segment and C-terminal segment of PsCRN63. Indicated plasmids combination were co-expressed in WT *Arabidopsis* protoplasts, subjected to a α-FLAG IP, and the bound protein was detected by immunoblot with the indicated antibodies.

(d) A sketch map for inverted association of PsCRN63. The dark grey end of the ribbon represents N-terminal of PsCRN63 and the shape of the explosion indicates where the association happens. The lower picture enlarges happened association as a schematic plot including combination sites. This diagrammatic sketch portrays a dimer association of PsCRN63.

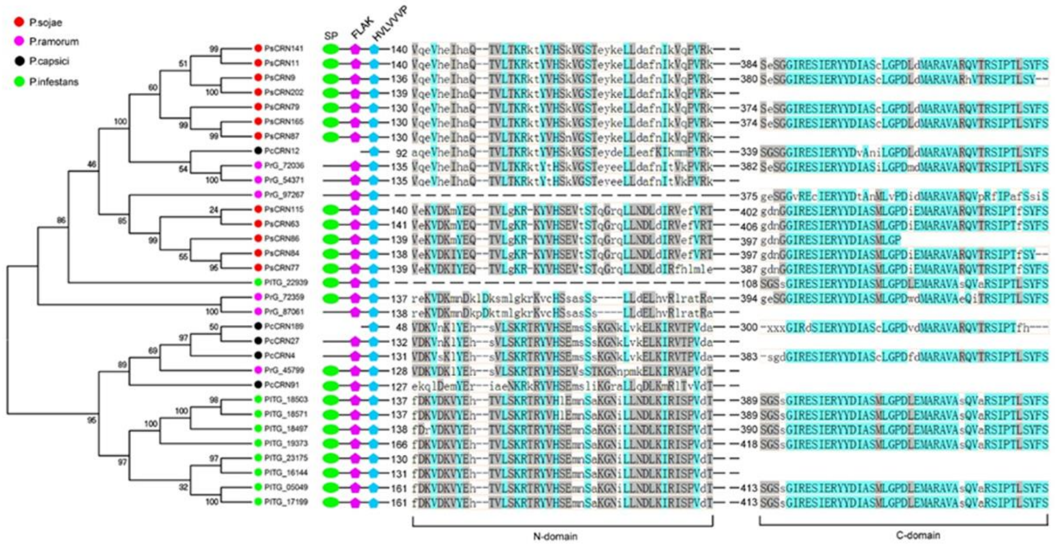

Supplementary Figure S5. Construction of phylogenetic tree for *Phytophthora* CRN effectors.

Thirty-two *Phytophthora* effectors were identified by similarity searches using BLAST analysis with an E-value cut-off 1E-10 in *P. sojae, P. ramorum, P. capsici* and *P. infestans* genome sequences. A phylogenetic tree of these effector proteins was constructed using MEGA 5.1 by the neighbor joining method. The predicted SP (Signal peptide), FLAK motif and HVLVVVP motif are indicated using corresponding geometric figure.

Supplementary Table S1. Suppression activities of flg22-induced *FRK1::LUC* expression of *Phytophthora sojae* effectors.

Effectors	relative *FRK1::LUC* expression	STDEV	Student's *t* test	Suppression of *FRK1::LUC*
Empty Vector	22.82	4.79		
HopAI1	3.23	0.47	0.01	YES
PsAvr1K	17.76	2.63	0.12	NO
PsAvr3C	15.74	6.06	0.15	NO
PsAvr4/6	24.50	6.65	0.27	NO
PsCRN63	2.11	0.48	0.02	YES
PsCRN115	23.38	2.28	0.84	NO
PsCRN124	17.38	5.53	0.75	NO
PsCRN127	21.73	0.48	0.28	NO

Supplementary Table S2. Oligonucleotides used for plasmids construction and gene expression.

**Primers for gene cloning**			
CRN63-XhoI-F	GCACTCGAGatgTTTCCTGTGGACATTGAC	CRN63-Csp45I-R	CGCTTCGAAGCTGAAATAACTAAACGTGGG
CRN115-XhoI-F	CATCTCGAGatgGCGTTCCCAGTGGACATC	CRN115-Csp45I-R	CGCTTCGAAGCTGAAATAACTAAACGTGGG
CRN127-XhoI-F	CATCTCGAGgtgTTTGACGTGGAAGTCGAC	CRN127-Csp45I-R	GCATTCGAA TAGCCTCACTTTCGTCAAAGA
ΔCRN63-2-XhoI-F	CATCTCGAGatgGAGACTTCCAAGATGGAT	CRN63:NES-Csp45I-R	CGCTTCGAACTTGTTAATATCAAGTCCAGC
ΔCRN63-3-XhoI-F	TATCTCGAGatgGTAACCTCGACCCAGGGC	CRN63:**nes**-Csp45I-R	GCATTCGAACTTGTTAGCATCTGCTCCAGC
		CRN63:NLS-Csp45I-R	CGCTTCGAACTTTACCTTTCGTTTCTTCTT
CRN63-NLS^{AAAA}-F	GAGGAGCAGCAGGCCAAAGCATATGCCGCATACGTGGAACAC	CRN63-NLS^{AAAA}-R	GTGTTCCACGTATGCGGCATATGCTTTGGCCTGCTGCTCCTC
CRN63:Δ43-F	ATACTCGAGatgGGCGCGTGGCTGCCAGAC	CRN63-N217-BstBI-R	GCATTCGAAGCCAATGTTGTGTTCCACGTA
CRN115:Δ43-F	ATACTCGAGatgGGCGCGTGGCTGCCAGAC	CRN115-N217-BstBI-R	GCATTCGAAGCCAATGTTGTGTTCCACGTA
CRN63-C45-XhoI-F	ATACTCGAGatgGGTGACAATGGCGGGATT	CRN63-N43-BstBI-R	ATATTCGAAGTCCGCCGTCTTCGCCAGGGA
CRN63-C83-XhoI-	ATACTCGAGatgCAGCCACCGACTGCACCC	CRN115-N43-BstBI-	ATATTCGAAGCCCTCCGTCTTCGCCAGGAA

F		R	
CRN63-C132-XhoI-F	ATACTCGAGatgGACCCAGTAATGGCGCTC	CRN63-N88-BstBI-R	GCATTCGAAGTTAGCTGTCAACACGTCCTC
CRN63-C177-XhoI-F	CATCTCGAGatgCTTGTTGCGATGAGGCCG	CRN63-N126-BstBI-R	CGCTTCGAAGAGTTGATCCATCTTGGAAGT
CRN63-C219-XhoI-F	ATACTCGAGatgGCTGTGCTCAAGGAGAAG	CRN63-N170-BstBI-R	GCATTCGAATGTGCGCACAAATTCCACCCG
CRN63-C266-XhoI-F	CTCGAGatgGTTCCATTCGATGCTGGA		
GFP-Csp45I-F	TCGTTCGAATACCCATACGATGTTCCTGAC	GFP-SpeI-R	CGAACTAGTTTACTTGTACAGCTCGTCCATGCC
CRN79-C45-XhoI-F	CATCTCGAGatgAGTGAAAGTGGCGGGATT	CRN79-N129-BstBI-R	CGCTTCGAACACTTGATCCATCCTGGAAGT
CRN79-C244-XhoI-F	ATACTCGAGatgGCTGCTGCTAAGTGGGGT	CRN79-N174-BstBI-R	CGCTTCGAATTTACGCACAGGTTGCACCTT
		CRN79-N221-BstBI-R	GCATTCGAAACCGAACACGCAAAGCTTCTC
CRN63-NcoI-F	GCACCATGGatgTTTCCTGTGGACATTGAC	CRN63-EcoRI-R	CGCGAATTCGCTGAAATAACTAAACGTGGG
CRN115-NcoI-F	ATACCATGGatgGACATCGATGGGGGCCAG	CRN115-EcoRI-R	CGCGAATTCGCTGAAATAACTAAACGTGGG
CRN63-	GCAGGTACCatgTTTCCTGTGGA	CRN63-	GCAGTCGACGCTGAAATAACTA

KpnI-F	CATTGAC	SalI-R	AACGTGGG
CRN115-KpnI-F	TATGGTACCatgGACATCGATGGGGGCCAG	CRN115-SalI-R	GCAGTCGACGCTGAAATAACTAAACGTGGG
PcCRN4-KpnI-F	CATGGTACCATGGTGAAGCTTTCACTACAG	PcCRN4-XhoI-R	GCACTCGAGACTGAAATAACTCAACGTGGG
PsCRN79-KpnI-F	GCAGGTACCATGGTCAAGCTTTTTTGTGCT	PsCRN79-SalI-R	GCAGTCGACGCTGAAATAACTCAACGTGGG
RxLR172-KpnI-F	tacctGGTACCATGTACAGCGGTGCAGTCGAGTC	RxLR172-SalI-R	TcgcGTCGACCCGCCCGTTGTCGCCCGTA
∆CRN63-2-KpnI-F	CATGGTACCatgGAGACTTCCAAGATGGAT	∆CRN63-3-KpnI-F	TATGGTACCatgGTAACCTCGACCCAGGGC

**Primers for RT-PCR**			
FRK1-F	GCCAACGGAGACATTAGAG	FRK1-R	CCATAACGACCTGACTCATC
NHL10-F	TTCCTGTCCGTAACCCAAAC	NHL10-R	CCCTCGTAGTAGGCATGAGC
WRKY53-F	CACCAGAGTCAAACCAGCCATTAC	WRKY53-R	CTTTACCATCATCAAGCCCATCGG
CBP60g-F	AAGAAGAATTGTCCGAGAGGAG	CBP60g-R	GGCGAGTTTATGAAGCACAG
PR1-F	GAAAACTTAGCCTGGGGTAGC	PR1-R	TTCATTAGTATGGCTTCTCGTTCA
PR2-F	GCAATGCAGAACATCGAGAA	PR2-R	TCATCCCTGAACCTTCCTTG
PDF1.2-F	CCATCATCACCCTTATCTTCGC	PDF1.2-R	TGTCCCACTTGGCTTCTCG
ERF1-F	TTAATTCAGTCCCCATTCTC	ERF1-R	CCAAGTCCCACTATTTTCAG
ORA59-F	CTCTGCTTCTACAATTTTTATG	ORA59-R	CTACACATCTATACATGTTTCC
ACT1-F	TCTTGATCTTGCTGGTCGTG	ACT1-R	GAGCTGGTTTTGGCTGTCTC
